# Predicting 90 day acute heart failure readmission and death using machine learning‐supported decision analysis

**DOI:** 10.1002/clc.23532

**Published:** 2020-12-23

**Authors:** FarnazBabaie Sarijaloo, Jaeyoung Park, Xiang Zhong, Anita Wokhlu

**Affiliations:** ^1^ Department of Industrial and Systems Engineering University of Florida Gainesville Florida USA; ^2^ Division of Cardiovascular Medicine University of Florida Gainesville Florida USA

**Keywords:** congestive heart failure, machine learning, mortality, repeat hospitalization

## Abstract

Readmission or death soon after heart failure (HF) admission is a significant problem. Traditional analyses for predicting such events often fail to consider the gamut of characteristics that may contribute– tending to focus on 30‐day outcomes even though the window of increased vulnerability may last up to 90 days. Risk assessments incorporating machine learning (ML) methods may be better suited than traditional statistical analyses alone to sort through multitude of data in the electronic health record (EHR) and identify patients at higher risk.

**Hypothesis:**

ML‐based decision analysis may better identify patients at increased risk for 90‐day acute HF readmission or death after incident HF admission.

**Methods and Results:**

Among 3189 patients who underwent index HF hospitalization, 15.2% experienced primary or acute HF readmission and 11.5% died within 90 days. For risk assessment models, 98 variables were considered across nine data categories. ML techniques were used to help select variables for a final logistic regression (LR) model. The final model's AUC was 0.760 (95% CI 0.752 to 0.767), with sensitivity of 83%. This proved superior to an LR model alone [AUC 0.744 (95% CI 0.732 to 0.755)]. Eighteen variables were identified as risk factors including dilated inferior vena cava, elevated blood pressure, elevated BUN, reduced albumin, abnormal sodium or bicarbonate, and NT pro‐BNP elevation. A risk prediction ML‐based model developed from comprehensive characteristics within the EHR can efficiently identify patients at elevated risk of 90‐day acute HF readmission or death for whom closer follow‐up or further interventions may be considered.

**Abbreviations**
AUC: area under the curve
BP: blood pressure
CI: confidence interval
EHR: electronic health record
HF: heart failure
ICD: international classification of diseases
LASSO: least absolute shrinkage and selection operator
LR: logistic regression
ML: machine learning
NT pro‐BNP: N‐terminal pro‐brain natriuretic peptide
OR: odds ratio
PPV: positive predictive value

## 1 INTRODUCTION

Heart failure is a global problem, affecting over 26 million people worldwide.^1^ In the United States alone, over 1 million people are hospitalized for a primary diagnosis of HF annually.^2^, ^3^ For these patients, readmission, or death in the post‐discharge phase after an incident HF admission is particularly problematic. Up to 25% of HF, patients may be readmitted within 30 days of discharge, with an additional mortality risk of ~10%.^4^, ^5^, ^6^, ^7^ In the past few years, multiple HF risk‐prediction models have been proposed to identify HF patients at highest risk of 30‐day readmission or death–with the focus on 30‐day outcomes, in part related to reporting standards and reimbursement penalties in the U.S.^8^, ^9^, ^10^, ^11^ However, window of vulnerability is likely longer, with the spike in event rates occurring out 90 days post‐discharge and plateauing thereafter.^5^, ^7^ Few existing HF risk prediction algorithms have explored readmission and death assessed over the post‐discharge phase transition out to 90 days.^12^, ^13^, ^14^

Another limitation of existing HF‐risk prediction models is the limited number of risk factors they assess. Compared to traditional statistical methodologies, machine learning (ML)‐supported techniques have the advantage of being able to cull through the information‐dense electronic health record (EHR) and to account for nonlinear interactions.^15^, ^16^ The EHR can be systematically mined not only for routine historical, laboratory, and medication data, but also for variables often lacking in other models, such as comprehensive echocardiographic data or socioeconomic contributors. However, the clinical interpretability of ML algorithms can vary. Combining ML techniques with more traditional statistical techniques like logistic regression (LR) has the potential advantage of greater interpretability, as well as increased performance.

The purpose of this study was to integrate multiple classes of risk factors found in the EHR into a comprehensive, ML‐based risk prediction model for 90‐day acute HF readmission or all‐cause mortality after incident HF admission.

## 2 METHODS

### 2.1 Patient population

This retrospective, single‐center study was approved by the University of Florida (UF) Institutional Review Board and Privacy Office as an exempt study with a waiver of informed consent. This study included 4368 consecutive patients with established outpatient primary care or cardiology care at UF Health Shands who subsequently underwent an incident HF hospitalization at UF Health Shands Hospital between January 2011 and January 2019 and survived to discharge. HF as a primary or secondary diagnosis was identified using ICD‐9 or ICD‐10 codes, listed in Supplemental Table 1. In addition to restricting the cohort to those serviced by our hospital system in outpatient care, it was restricted further to those living in the UF‐Shands primary service area in order to limit the potential for outside hospitalizations. In addition, 1179 patients were excluded from the study because of lack of recent echocardiographic data prior to discharge or insufficient vital information, resulting in 3189 patients in the analysis cohort.

### 2.2 Outcomes

The primary outcome was defined as readmission due to acute HF or all‐cause mortality within 90 days after discharge from an index HF hospitalization. Readmission was attributed to acute HF if any of the following occurred: (a) readmission with a primary HF diagnosis, (b) readmission with a secondary HF diagnosis denoting acuity (i.e. ICD‐10 [ICD‐9 where applicable] codes: I50.21 [428.21], I50.23 [428.23], I50.31 [428.31], I50.33 [428.33], I50.41 [428.41], I50.43 [428.43], I50.811 and I50.813), or (c) readmission with a secondary HF diagnostic code of any acuity along with receipt of intravenous diuretics during hospitalization.

### 2.3 Data preprocessing

Data were extracted from the EPIC EHR and the McKesson Change echocardiography reporting system. Variables were collected among nine categories: (a) demographic and socioeconomic characteristics, (b) outpatient care characteristics, (c) social history including lifestyle and noncompliance behavior, (d) medical history, (e) hospitalization characteristics, (f) laboratory values including cardiac biomarkers, (g) vitals, (h) medications, and (i) comprehensive transthoracic echocardiographic findings. In cases where echocardiographic reports were incomplete for certain data elements, an experienced echocardiographer provided additional review. The criteria used were as outlined in various American Society of Echocardiography guidelines.^17^, ^18^ The presence of pulmonary hypertension on echocardiography was defined as right ventricular systolic pressure ≥ 35 mmHg or tricuspid regurgitant velocity ≥ 2.7 m/s, in comparison to normal or absent values.

Overall, 141 variables were considered, but 34 variables with ≥50.0% missing data and 9 variables with purity >99.0% were removed. The final design matrix had 98 variables. Among them, 89 variables had no missing data, 5 variables had <1.0% missing data, 1 variable (Troponin T) had 19.6% missing data, and 3 variables (albumin, bicarbonate, and N‐terminal pro‐brain natriuretic peptide [NT pro‐BNP]) had between 32.7% to 47.7% missing data. Missing laboratory values were imputed using re‐sampling based on readmission status. In order to model nonlinear effects, some continuous variables were transformed into categorical variables.

### 2.4 Variable selection

**FIGURE 1:**
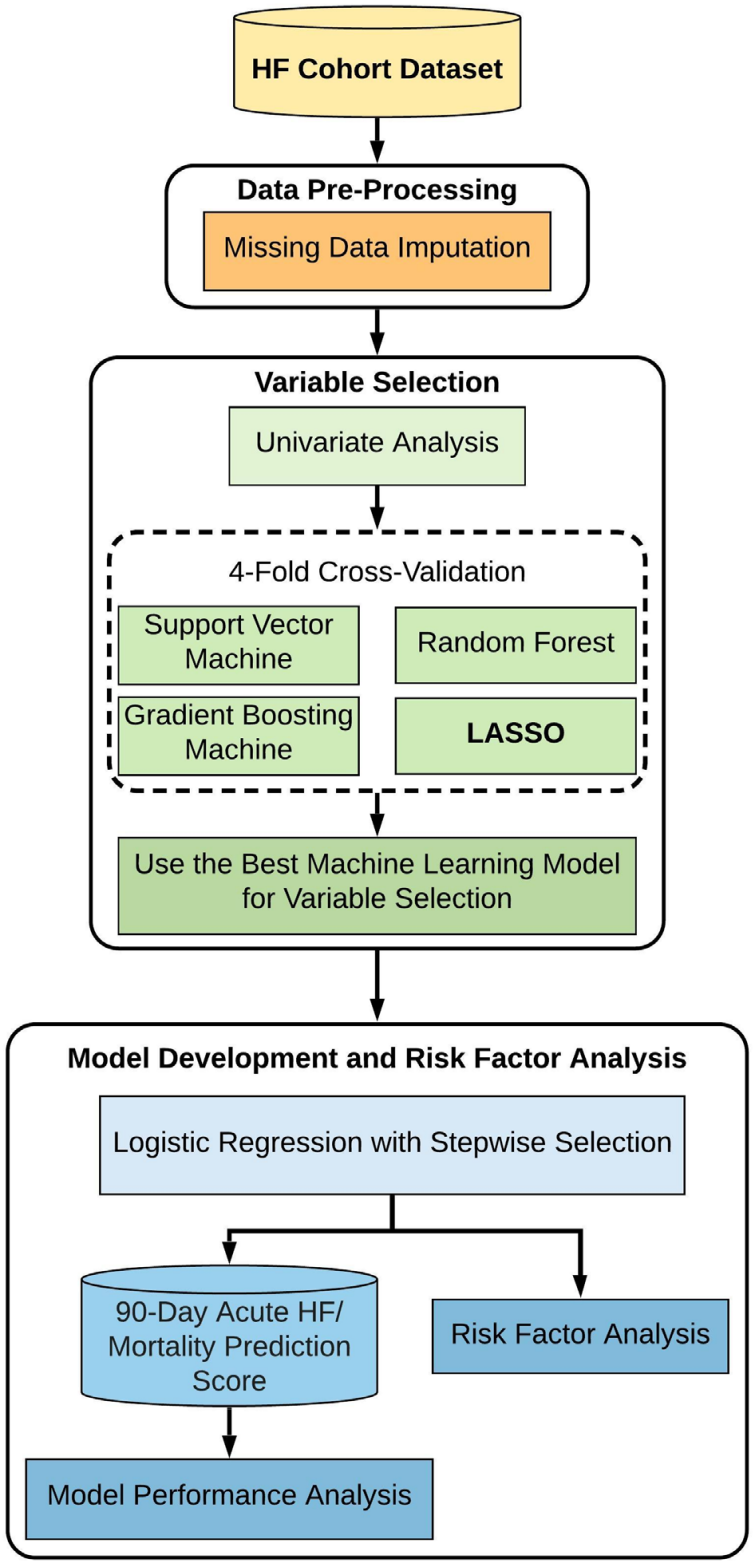
Flow diagram of 90‐day heart failure readmission and mortality risk prediction model development using machine learning techniques

Figure 1 is a flow diagram highlighting the variable selection process for the risk model. Univariate analysis was performed to determine which factors were significantly associated with 90‐day acute HF readmission or all‐cause mortality. Specifically, unpaired categorical variables were compared between subgroups using chi‐square test. Continuous variables were assessed using t‐test. Candidate univariates with *p* < .10 were selected for inclusion in ML‐based models for variable selection. These variables were also used to develop an LR‐only model as the benchmark for performance comparison. Area under the curve (AUC) was used as the performance metrics.

For ML‐based variable selection, the entire cohort was split randomly into 4‐folds. Then, 4‐fold cross‐validation was performed in which each fold (25% of the dataset) was selected consecutively as a testing dataset and the remaining folds (75%) were combined into a training dataset. Subsequently, four ML algorithms, which are typically, used in this field,^19^, ^20^, ^21^ including (a) least absolute shrinkage and selection operator (LASSO), (b) gradient boosting machine, (c) random forest, and (d) support vector machine were applied. During this process, the parameters were optimized for each model by maximizing AUC. Using the best performing model, variables observed to be significant (*p* < .10) in at least 3‐folds were selected for further inclusion into an LR model for interpretable risk factor analysis, which is named as a combined ML‐LR model.

### 2.5 Model development and performance analysis

A final combined ML‐LR model with stepwise selection based on Akaike Information Criterion was developed to predict the risk of the 90‐day acute HF combined endpoint. Final model performance was assessed using AUC. To classify patients, a discrimination threshold, that is, a cutoff value within the model for elevated 90‐day HF risk, was assessed. Patients with a 90‐day acute HF prediction value above the discrimination threshold were labeled as at high risk of readmission or death. The threshold was calculated by either (a) simultaneously optimizing the sensitivity and specificity of the model using Youden's J‐statistic or (b) minimizing weighted total mis‐specification cost (by defining a relative cost in terms of a ratio between false negative and false positive cases).

### 2.6 Risk factor analysis

To identify risk factors of 90‐day acute HF readmission or death, relative risks were expressed as odds ratios (OR) with 95% confidence intervals (CI). Those with a *p* < .05 were considered significant. In addition, sensitivity analyses were performed to resample any variables with >20% imputation and to identify any impact on AUC characteristics.

### 2.7 Software

Analyses were performed using R^22^ version 3.6.0. and R packages including randomForest,^23^ gbm,^24^ glmnet^25^ and e1071.^26^

## 3 RESULTS

### 3.1 Patient characteristics

In the cohort, more than half of all the patients in the study were women and almost one‐third were non‐white. Mean age was 67.8 years with a standard deviation of 15.0. Coronary artery disease was present in 61% of the cohort. Mean left ventricular ejection fraction was 48.4% with a standard deviation of 16.6%. Among the overall cohort, 58% presented with acute HF. The mean length of stay was 7.2 days with a standard deviation of 13.0.

### 3.2 Outcomes

Among the 3189 patients in the cohort, 485 (15.2%) were readmitted because of acute HF and 367 (11.5%) died within 90 days after discharge, resulting in 790 (24.8%) who experienced the 90‐day composite endpoint and 2399 patients (75.2%) who did not. The mean time to event was 28 days. Among the 790 patients who experienced HF readmission or death, 412 (52.2%) experienced their first readmission or death early, that is, within the first 30 days after discharge—among these patients, 302 (73.3%) had their first acute HF readmission and 110 (26.7%) died without readmission. Among 378 patients (47.8%) who experienced their first event in a delayed fashion, that is, within 31 to 90 days, the distribution of outcome tended toward more hospitalization events‐ with 319 (84.4%) of them having their first acute HF readmission and 59 (15.6%) dying without readmission.

### 3.3 Univariate analysis of 90‐day acute HF readmission or death

Supplemental Table 2 compares the baseline characteristics of patients who experienced 90‐day acute HF readmission or death with those who did not, identifying numerous differences across categories. In the univariate analysis, 66 variables with a significance level of 0.10 were identified for inclusion into an LR‐only model (for comparison) as well as ML models for further variable selection.

### 3.4 Machine learning to complete variable selection

Table 1 demonstrates the performance of the various ML models used for variable selection and LR‐only model without ML. Among the four distinct ML models, the LASSO (AUC 0.748, 95% CI 0.745–0.751) and gradient boosting machine models (0.750, 95% CI 0.743–0.756) were better than other ML algorithms. The LASSO and gradient‐boosting models were comparable and demonstrated substantial overlap in predictor variables. Due to the similarity in performance and the large overlap in variables selected, the LASSO model, which was felt to be more clinically interpretable, was used to complete variable selection, identifying 43 relevant variables for inclusion into a final combined ML‐LR model. We tested other variable selection methods, e.g., using the variables selected by gradient‐boosting models, or using the overlapped variables selected by both the LASSO and gradient‐boosting models, and the performance of these combined ML‐LR models were slightly inferior to the one using LASSO.

**TABLE 1:** Comparison of Machine Learning and Logistic Regression Models

Model	AUC	95% CI	Comments
LR Only	0.744	(0.732–0.755)	Comparator model
ML‐LASSO only	0.748	(0.745–0.751)	Variable selection option
ML‐GBM only	0.750	(0.743–0.756)	Variable selection option
ML‐ RF only	0.570	(0.545–0.594)	Variable selection option
ML‐SVM only	0.718	(0.703–0.733)	Variable selection option
Combined ML‐LASSO + LR	0.760	(0.752–0.767)	Final combined model

Abbreviations: AUC, area under the curve; CI, confidence interval; GBM, gradient boosting machine; LASSO, least absolute shrinkage and selection operator; LR, logistic regression; RF, random forest; SVM, support vector machine.

### 3.5 Final risk assessment model

**FIGURE 2:**
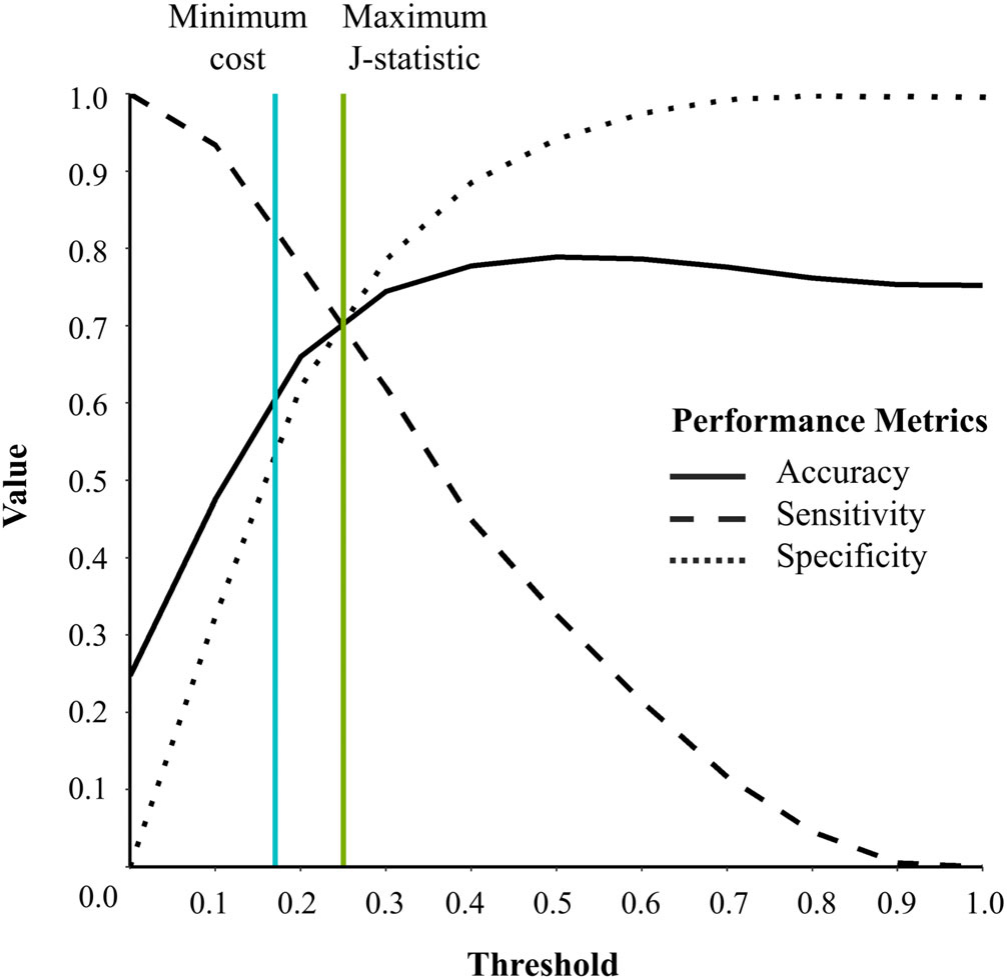
Performance metrics of the combined final 90‐day risk model. The final combined LASSO‐LR model had an AUC of 0.760 (95% CI 0.752 to 0.767). Using a threshold to maximize the Youden's J statistic (green line), the model demonstrated a sensitivity of 70%, a specificity of 71%, and accuracy of 71%. Using a lower threshold to minimize cost of false positives and false negatives (blue line) yielded a higher sensitivity but reduced accuracy

The final model had an AUC of 0.760 (95% CI 0.752 to 0.767). Random sampling of variables with >20% missing values did not affect the overall performance of the model (average AUC was 0.759, 95% CI 0.758 to 0.760). As shown in Figure 2, in the decision rule analysis, adjusting the threshold to maximize the Youden's J statistic yielded a sensitivity of 70%, a specificity of 71%, and an accuracy of 71%, with a positive predictive value of 45%. Applying a lower threshold to minimize the mis‐specification cost resulted in a higher sensitivity of 83%, but a reduced specificity (56%), accuracy (63%) and positive predictive value (38%).

### 3.6 90‐day acute HF or death risk factors

**FIGURE 3:**
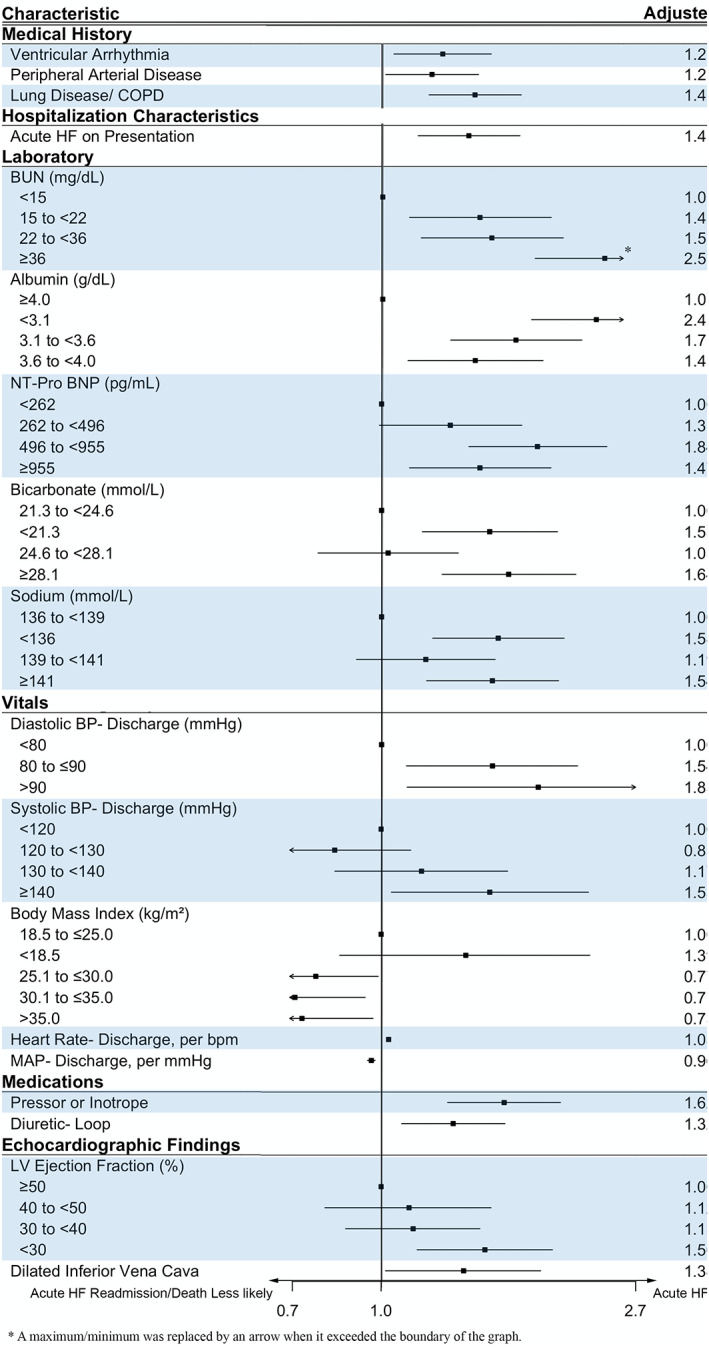
Forest plot of the risk factors associated with 90‐day acute heart failure or death. Significant characteristic categories are shown (*p* < .05). Adjusted odds ratio (OR) is displayed with 95% confidence interval (CI). BP, blood pressure; bpm, beats per minute; BUN, blood urea nitrogen; COPD, chronic obstructive pulmonary disease; LV, left ventricular; MAP, mean arterial pressure; NT pro‐BNP, Nterminal pro‐brain natriuretic peptide

Twenty‐five putative risk factors of acute HF readmission or death at 90 days were identified, of which 18 were significant with a *p* < .05. Figure 3 demonstrates the OR associated with each significant risk factor. The strongest risk factors, with an OR of at least 1.5 and *p* < .05, were in the laboratory value category: low albumin levels, elevated NT pro‐BNP > 496 pg/mL, and the extremes of bicarbonate and sodium levels. Discharge vitals were also highly significant; elevated systolic BP ≥140 mmHg, elevated diastolic BP≥80 mmHg, and increasing heart rate were associated with increased risk of 90‐day HF events. The main comorbidities associated with adverse HF outcome were lung disease or other cardiovascular diagnoses. The analysis further demonstrated that patients who present with acute HF have a higher risk of readmission or death than patients who present less acutely. Similarly, dilated inferior vena cava, an echocardiographic sign of overt volume overload; loop diuretic administration; severe LV dysfunction, and the need for inotropic support portended worse outcomes.

## 4 DISCUSSION

In this study, using data derived from a standard EHR, advanced ML techniques were employed to create a comprehensive combined ML‐LR prediction model of 90‐day HF readmission or all‐cause death after an index HF admission. Previous models of HF risk typically have focused on a limited set of predictors. In this moderately predictive model, the identification of 18 risk factors underscores the inherent complexity of risk prediction and the important role for such models in supplementing clinical judgment. In addition, in this cohort, almost one‐third experienced their first clinical endpoint within 30 to 90 days after discharge; supporting the notion that this longer window of vulnerability may be relevant.^5^, ^7^

### 4.1 90‐day HF model performance

We are not aware of any other models looking at 90‐day HF readmission and death. A previous EVEREST analysis provided a comprehensive description of various factors in the course of HF patients who experience 90‐day all‐cause rehospitalization or death after discharge. ^12^ In addition, another moderately predictive model (AUC 0.730) looking at 90‐day all‐cause readmission identified the Charleston comorbidity index, NT pro‐BNP, and certain hematologic parameters as predictors in a Chinese cohort of HF patients after discharge.^13^

The current model appears more discriminative with an AUC of 0.760 but its performance requires further validation. Although direct comparison was not performed, performance characteristics appear comparable to commonly accepted 30‐day HF models but provides a longer outlook in the vulnerable window post‐discharge. For comparison, existing 30‐day models report AUCs ranging from 0.55 to 0.83.^12^ Among them, the Yale Readmission Risk Score, the most widely used risk prediction model for 30‐day HF readmission, demonstrates modest predictive ability with an AUC in the range of 0.60 to 0.61.^18^, ^27^ The current 90‐day model shares some common characteristics with various 30‐day models including acute HF admission, lung disease, BP, heart rate, sodium, NT pro‐BNP, and left ventricular ejection fraction. However, this model has some distinct elements.^28^, ^29^

### 4.2 The Gamut of 90‐day HF predictors

The evaluation of echocardiographic parameters was one unique aspect of this model. Despite assessing multiple parameters, only left ventricular ejection fraction <30% and dilated inferior vena cava were significantly associated with the 90‐day HF composite outcome. This finding meshes with a previous observation that dilated inferior vena cava augments the performance of the Yale CORE 30‐day HF readmission score.^30^, ^31^ In addition, akin to findings from EVEREST, vitals, were robustly associated with 90‐day HF events. Elevated BP and heart rate parameters likely reflect the adequacy of pharmacological treatment and serve as metrics of decompensation.

**FIGURE 4:**
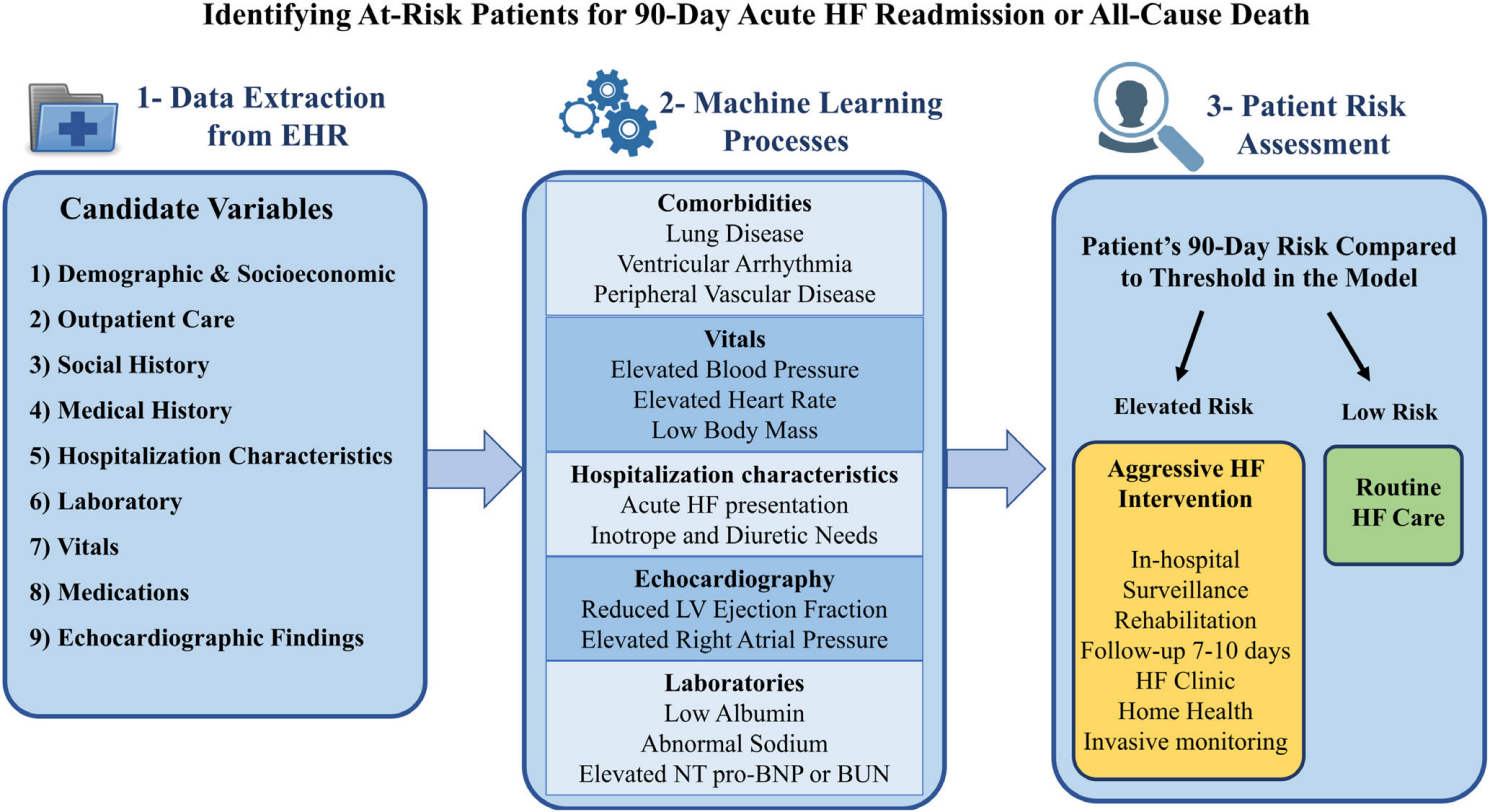
Identifying at‐risk patients for 90‐day acute heart failure readmission or all‐cause death. A schematic overview of how machine learning algorithms can be used to integrate multiple characteristics into a comprehensive risk prediction model for 90‐day heart failure (HF) readmission or all‐cause death after discharge from an index admission. Patients identified as greater risk during the 90‐day window of vulnerability may receive additional attention and clinical intervention. BUN, blood urea nitrogen; LV, left ventricular; NT pro‐BNP, N‐terminal pro‐brain natriuretic peptide

Socioeconomic factors and compliance documentation, interestingly, were not identified as significant mediators of risk. This may be because other factors such as laboratory parameters were so strongly associated. Several laboratory abnormalities, including sodium, those related to renal function, and NT pro‐BNP were important. Although some laboratories have been identified previously as significant 30‐day predictors, others like NT pro‐BNP had yet to be incorporated in such comprehensive longer outlook models.^32^ Figure 4 summarizes how ML‐based processes can be used to identify HF patients at elevated risk at discharge.

### 4.3 Limitations

One limitation of this study is that it is a single‐center retrospective study. Therefore, it has less applicability than existing 30‐day models, like the Yale Readmission Score, which have been extensively validated in Medicare recipients. The current model would benefit from validation in a larger multi‐center or prospective cohort. Another limitation is that the applicability of this model to patients without baseline echocardiography was not assessed. Similarly, laboratory variables had missing data. Although standard methods for imputing data were employed, variables such as biomarkers that may be ordered less often might be missed in the risk prediction process.

## 5 CONCLUSION

The current study supports a role for the assimilating ML‐based risk assessment into clinical care of HF patients after index admission to identify who is high risk. Such combined ML risk assessment is broadly applicable to care of HF patients, particularly in the era of common EHRs. The same technique of data extraction and variable selection can be applied to any institution or cohort, allowing for the model to be revalidated over time to reflect a dynamic population. Ultimately, the goal of predictive models like this one is to identify vulnerable HF patients and to take actions in the post‐discharge period that can reduce future HF risk.

## CONFLICT OF INTEREST

Dr Zhong and Dr Wokhlu received research funding support with a sub‐grant to University of Florida from University of Wisconsin, funded by Baxter Healthcare. They (Xiang Zhong, Anita Wokhlu) take full responsibility for the preparation of the manuscript and the decision to publish

## Supporting information


**Supplemental Table 1** ICD‐10 and ICD‐9 codes used for heart failure diagnosis

**Supplemental Table 2** Characteristics based on 90‐day acute heart failure readmission or all‐cause death

## Data Availability

The data that support the findings of this study may be available on request from the corresponding author, but will require institutional permission and notification of the sponsor. The data are not publicly available and access may be further restricted due to privacy or ethical restrictions.
