# Supplementary material for: Predicting 90 day acute heart failure readmission and death using machine learning‐supported decision analysis
Source: Clin Cardiol. 2020 Dec 23;44(2):230–7. doi: 10.1002/clc.23532 (PMC7852168; doi:10.1002/clc.23532)
Supplement: Supplementary file 1 — Supplemental Table 1 ICD‐10 and ICD‐9 codes used for heart failure diagnosis [file CLC-44-230-s001.docx]

**Supplemental Table 1. ICD-10 and ICD-9 codes used for heart failure diagnosis**

| **ICD-10 (ICD-9) codes** | **Diagnosis** |
| --- | --- |
| I50 (428.0) | HF |
| I50.1 (428.1) | Left ventricular failure, unspecified |
| I50.2 (428.2) | Systolic (congestive) HF |
| I50.20 (428.20) | Unspecified systolic (congestive) HF |
| I50.21 (428.21) | Acute systolic (congestive) HF |
| I50.22 (428.22) | Chronic systolic (congestive) HF |
| I50.23 (428.23) | Acute on chronic systolic (congestive) HF |
| I50.3 | Diastolic (congestive) HF |
| I50.30 (428.30) | Unspecified diastolic (congestive) HF |
| I50.31 (428.31) | Acute diastolic (congestive) HF |
| I50.32 (428.32) | Chronic diastolic (congestive) HF |
| I50.33 (428.33) | Acute on chronic diastolic (congestive) HF |
| I50.4 | Combined systolic (congestive) and diastolic (congestive) HF |
| I50.40 (428.40) | Unspecified combined systolic (congestive) and diastolic (congestive) HF |
| I50.41 (428.41) | Acute combined systolic (congestive) and diastolic (congestive) HF |
| I50.42 (428.42) | Chronic combined systolic (congestive) and diastolic (congestive) HF |
| I50.43 (428.43) | Acute on chronic combined systolic (congestive) and diastolic (congestive) HF |
| I50.8 (428.9) | Other HF |
| I50.810 | Right HF |
| I50.811 | Acute right HF |
| I50.812 | Chronic right HF |
| I50.813 | Acute on chronic right HF |
| I50.814 | Right HF due to left HF |
| I50.82 | Biventricular HF |
| I50.83 | High output HF |
| I50.84 | End stage HF |
| I50.89 | Other HF |
| I50.9 (428.9) | HF, unspecified |
| R57.0 (785.51) | Cardiogenic shock |
| I11.0 (402.01, 402.11, 402.91) | Hypertensive heart disease with HF |

HF= heart failure; ICD= international classification of diseases.
