# Supplementary material for: Predicting 90 day acute heart failure readmission and death using machine learning‐supported decision analysis
Source: Clin Cardiol. 2020 Dec 23;44(2):230–7. doi: 10.1002/clc.23532 (PMC7852168; doi:10.1002/clc.23532)
Supplement: Supplementary file 2 — Supplemental Table 2 Characteristics based on 90‐day acute heart failure readmission or all‐cause death [file CLC-44-230-s002.docx]

**Supplemental Table 2. Characteristics based on 90-day acute heart failure readmission or all-cause death**

| **Characteristic** | **90-day HF event**  **(N=790)** | **No 90-day HF event**  **(N=2399)** | **P-value** |
| --- | --- | --- | --- |
| **Demographic & Socioeconomic** | | | |
| Age, yr. | 70.5± 14.4 | 67.1±15.1 | **<0.001** |
| Male sex | 363 (45.9) | 1106 (46.1) | 0.973 |
| Race |  |  | 0.464 |
| *Black* | 222 (28.1) | 729 (30.4) |  |
| *White* | 536 (67.8) | 1580 (65.9) |  |
| *Other* | 32 (4.1) | 90 (3.8) |  |
| Married | 302 (38.2) | 957 (39.9) | 0.431 |
| Rural Resident | 88 (11.1) | 285 (11.9) | 0.618 |
| Insurance |  |  | **<0.001** |
| *Medicare* | 598 (75.7) | 1621 (67.6) |  |
| *Medicaid* | 84 (10.6) | 247 (10.3) |  |
| *Private/Managed Care* | 18 (2.3) | 99 (4.1) |  |
| *Other* | 90 (11.4) | 432 (18.0) |  |
| **Outpatient Care** | | | |
| Electronic Health Portal Use | 169 (21.4) | 595 (24.8) | **0.057** |
| No. Cardiology Visits in 1-Year |  |  | **<0.001** |
| *0* | 598 (75.7) | 1628 (67.9) |  |
| *1 to 2* | 115 (14.6) | 431 (18.0) |  |
| *≥3* | 77 (9.7) | 340 (14.2) |  |
| No. No-Shows in 1-Year  *0* | 642 (81.3) | 2002 (83.5) | 0.114 |
| *1 to 2* | 101 (12.8) | 304 (12.7) |  |
| *≥3* | 47 (5.9) | 93 (3.9) |  |
| **Social History** | | | |
| Tobacco Abuse/Smoking | 448 (56.7) | 1303 (54.3) | 0.258 |
| Alcohol Dependence | 86 (10.9) | 259 (10.8) | 0.996 |
| Illicit Drug Use | 59 (7.5) | 213 (8.9) | 0.247 |
| Noncompliance | 244 (30.9) | 730 (30.4) | 0.843 |
| **Medical History (Cardiovascular-related)** | | | |
| Hypertension | 728 (92.2) | 2241 (93.4) | 0.257 |
| Dyslipidemia | 596 (75.4) | 1813 (75.6) | 0.979 |
| Pulmonary Hypertension | 75 (9.5) | 178 (7.4) | **0.073** |
| Cardiomyopathy Diagnosis | 312 (39.5) | 930 (38.8) | 0.748 |
| Congestive Heart Failure | 456 (57.7) | 1337 (55.7) | 0.350 |
| Coronary Artery Disease | 476 (60.3) | 1461 (60.9) | 0.779 |
| Myocardial Infarction | 251 (31.8) | 653 (27.2) | **0.016** |
| CABG or PCI | 392 (49.6) | 1129 (47.1) | 0.227 |
| Stroke or TIA | 455 (57.6) | 1312 (54.7) | 0.166 |
| Pacemaker or Defibrillator | 74 (9.4) | 222 (9.3) | 0.980 |
| Valvular Heart Disease | 299 (37.8) | 789 (32.9) | **0.012** |
| Atrial Arrhythmia | 438 (55.4) | 1083 (45.1) | **<0.001** |
| Ventricular Arrhythmia | 514 (65.1) | 1371 (57.1) | **<0.001** |
| Peripheral Arterial Disease | 267 (33.8) | 634 (26.4) | **<0.001** |
| **Medical History (Other)** |  |  |  |
| Moderate/ Severe Renal Disease | 264 (33.4) | 693 (28.9) | **0.018** |
| Malignancy/ Cancer | 499 (63.2) | 1451 (60.5) | 0.194 |
| Depression/ Psychiatric | 451 (57.1) | 1355 (56.5) | 0.797 |
| Cognitive Dysfunction | 166 (21.1) | 404 (16.8) | **0.009** |
| Diabetes | 411 (52.0) | 1270 (52.9) | 0.685 |
| Endocrine- Thyroid Diseases | 250 (31.6) | 821 (34.2) | 0.198 |
| Hypogonadism | 67 (8.5) | 240 (10.0) | 0.234 |
| Venous Thromboembolism | 132 (16.7) | 321 (13.4) | **0.023** |
| Liver Disease | 247 (31.3) | 601 (25.1) | **<0.001** |
| Sleep Apnea | 275 (34.8) | 958 (39.9) | **0.012** |
| Lung Disease/ COPD | 486 (61.9) | 1277 (53.2) | **<0.001** |
| Chronic Oxygen Use | 128 (16.2) | 292 (12.2) | **0.004** |
| **Hospitalization Characteristics** |  |  |  |
| Acute HF on Presentation | 561 (71.0) | 1282 (53.4) | **<0.001** |
| Length of Stay (days) |  |  | **<0.001** |
| *≤2* | 108 (13.7) | 516 (21.5) |  |
| *3 to 4* | 153 (19.4) | 684 (28.5) |  |
| *5 to 8* | 229 (29.0) | 659 (27.5) |  |
| *≥9* | 300 (38.0) | 540 (22.5) |  |
| Observation Status | 20 (2.5) | 223 (9.3) | **<0.001** |
| Intensive Care Unit | 247 (31.3) | 484 (20.2) | **<0.001** |
| Discharge Med Reconciliation | 90 (11.4) | 251 (10.5) | 0.505 |
| **Laboratory** |  |  |  |
| Albumin (g/dL) |  |  | **<0.001** |
| *<3.1* | 269 (34.1) | 500 (20.9) |  |
| *3.1 to <3.6* | 219 (27.8) | 591 (24.7) |  |
| *3.6 to <4.0* | 172 (21.8) | 635 (26.5) |  |
| *≥4.0* | 130 (16.5) | 673 (28.1) |  |
| Bicarbonate (mmol/L**)** |  |  | **0.008** |
| *<21.3* | 233 (29.5) | 527 (22.0) |  |
| *21.3 to <24.6* | 161 (20.4) | 589 (24.6) |  |
| *24.6 to <28.1* | 159 (20.2) | 632 (26.4) |  |
| *≥28.1* | 237 (30.0) | 651 (27.2) |  |
| BUN (mg/dL) |  |  | **<0.001** |
| *<15* | 109 (13.9) | 657 (27.4) |  |
| *15 to <22* | 171 (21.7) | 657 (27.4) |  |
| *22 to <36* | 198 (25.1) | 582 (24.3) |  |
| *≥36* | 312 (39.5) | 503 (21.0) |  |
| Creatinine (mg/dL) |  |  | **<0.001** |
| *<0.8* | 173 (21.9) | 597 (24.9) |  |
| *0.8 to <1.1* | 138 (17.5) | 673 (28.1) |  |
| *1.1 to <1.7* | 209 (26.5) | 594 (24.8) |  |
| *≥1.7* | 270 (34.2) | 535 (22.3) |  |
| Hemoglobin (g/dL) |  |  | **<0.001** |
| *<8.5* | 276 (35.0) | 525 (21.9) |  |
| *8.5 to <10.0* | 219 (27.8) | 575 (24.0) |  |
| *10.0 to <12.0* | 161 (20.4) | 628 (26.2) |  |
| *≥12.0* | 134 (17.0) | 671 (28.0) |  |
| NT pro-BNP (pg/mL) |  |  | **<0.001** |
| *<262* | 123 (15.6) | 693 (28.9) |  |
| *262 to <496* | 195 (24.7) | 595 (24.8) |  |
| *496 to <955* | 262 (33.2) | 542 (22.6) |  |
| *≥955* | 210 (26.6) | 569 (23.8) |  |
| Sodium (mmol/L) |  |  | **<0.001** |
| *<136* | 235 (29.8) | 529 (22.1) |  |
| *136 to <139* | 163 (20.7) | 658 (27.5) |  |
| *139 to <141* | 159 (20.2) | 620 (25.9) |  |
| *≥141* | 233 (29.5) | 592 (24.7) |  |
| Troponin T (ng/mL) |  |  | **<0.001** |
| *<0.03* | 105 (13.3) | 386 (16.1) |  |
| *0.03 to <0.15* | 381 (48.3) | 1435 (59.8) |  |
| *≥0.15* | 304 (38.5) | 578 (24.1) |  |
| **Vitals** |  |  |  |
| Body Mass Index (kg/m^2^) |  |  | **<0.001** |
| *<18.5* | 39 (5.0) | 59 (2.5) |  |
| *18.5 to* ≤ *25.0* | 253 (32.1) | 552 (23.1) |  |
| *25.1 to* ≤*30.0* | 211 (26.8) | 650 (27.1) |  |
| *30.1 to* ≤*35.0* | 137 (17.4) | 507 (21.2) |  |
| *>35.0* | 150 (19.0) | 631 (26.4) |  |
| Weight, kg |  |  |  |
| Weight- Outpatient | 84.1±27.0 | 89.1±27.7 | **<0.001** |
| Weight- Admission | 83.0±27.9 | 88.6±28.0 | **<0.001** |
| Weight Gain at Presentation | -1.1±7.1 | -0.5±7.1 | **0.033** |
| Weight -Maximum Inpatient | 87.4±28.8 | 91.7±28.8 | **<0.001** |
| Weight- Minimum Inpatient | 79 ±26.6 | 85.2±27.3 | **<0.001** |
| Weight Loss Over Hospitalization | 8.3±8.6 | 6.4±7.8 | **<0.001** |
| Weight- Discharge | 82.8±27.7 | 87.9±27.9 | **<0.001** |
| Blood Pressure (mmHg) |  |  |  |
| Systolic BP- Admission |  |  | **<0.001** |
| *<120* | 282 (35.7) | 641 (26.7) |  |
| *120 to <130* | 119 (15.1) | 362 (15.1) |  |
| *130 to <140* | 118 (14.9) | 410 (17.1) |  |
| *≥140* | 271 (34.3) | 986 (41.1) |  |
| Systolic BP- Discharge |  |  | **<0.001** |
| *<120* | 398 (50.4) | 886 (36.9) |  |
| *120 to <130* | 108 (13.7) | 429 (17.9) |  |
| *130 to <140* | 101 (12.8) | 414 (17.3) |  |
| *≥140* | 183 (23.2) | 670 (27.9) |  |
| Diastolic BP- Admission |  |  | **0.054** |
| *<80* | 584 (73.9) | 1671 (69.7) |  |
| *80 to* ≤*90* | 118 (14.9) | 394 (16.4) |  |
| *>90* | 88 (11.1) | 334 (13.9) |  |
| Diastolic BP- Discharge |  |  | **0.008** |
| *<80* | 661 (83.7) | 1884 (78.5) |  |
| *80 to* ≤*90* | 89 (11.3) | 359 (15.0) |  |
| *>90* | 40 (5.1) | 156 (6.5) |  |
| Pulse Pressure- Admission |  |  | **<0.001** |
| *<40* | 132 (16.7) | 250 (10.4) |  |
| *40 to* ≤*60* | 306 (38.7) | 954 (39.8) |  |
| *61 to* ≤*80* | 239 (30.3) | 782 (32.6) |  |
| *>80* | 113 (14.3) | 413 (17.2) |  |
| Pulse Pressure- Discharge |  |  | **<0.001** |
| *<40* | 167 (21.1) | 361 (15.0) |  |
| *40 to* ≤*60* | 334 (42.3) | 1088 (45.4) |  |
| *61 to* ≤*80* | 219 (27.7) | 736 (30.7) |  |
| *>80* | 70 (8.9) | 214 (8.9) |  |
| MAP- Admission, mmHg | 91.2±15.4 | 94.4 ±15.5 | **<0.001** |
| MAP- Discharge, mmHg | 85.4±13.7 | 89.8±12.0 | **<0.001** |
| Heart Rate- Admission, bpm | 88.3±18.4 | 83.9±17.2 | **<0.001** |
| Heart Rate- Discharge, bpm | 81.7±16.0 | 76±12.3 | **<0.001** |
| **Medications** | | | |
| Aspirin | 576 (72.9) | 1767 (73.7) | 0.715 |
| ACE-I/ ARB/ ARNI | 464 (58.7) | 1595 (66.5) | **0.001** |
| Aldosterone Antagonists | 60 (7.6) | 176 (7.3) | 0.871 |
| Beta Blocker | 684 (86.6) | 1981 (82.6) | **0.01** |
| Antiarrhythmics | 153 (19.4) | 291 (12.1) | **<0.001** |
| Anticoagulation | 215 (27.2) | 601 (25.1) | 0.245 |
| Ca-Channel Blocker (CCB), any | 232 (29.4) | 786 (32.8) | **0.083** |
| CCB, non-dihydropyridine | 170 (21.5) | 383 (16.0) | **<0.001** |
| Digoxin | 69 (8.7) | 99 (4.1) | **<0.001** |
| Diuretic- Metolazone | 88 (11.1) | 103 (4.3) | **<0.001** |
| Diuretic- Loop | 483 (61.1) | 1236 (51.5) | **<0.001** |
| Diuretic- Thiazide | 98 (12.4) | 372 (15.5) | **0.038** |
| Pressor or Inotrope | 266 (33.7) | 442 (18.4) | **<0.001** |
| Nitrate | 112 (14.2) | 288 (12.0) | 0.124 |
| Hydralazine | 227 (28.7) | 697 (29.1) | 0.899 |
| Statin | 498 (63.0) | 1539 (64.2) | 0.601 |
| Insulin | 426 (3.9) | 1101 (45.9) | **<0.001** |
| Metformin | 49 (6.2) | 195 (8.1) | **0.091** |
| Any Estrogen | 10 (1.3) | 39 (1.6) | 0.585 |
| NSAID | 104 (13.2) | 363 (15.1) | 0.194 |
| **Echocardiographic Findings** |  |  |  |
| Dilated LV | 113 (14.3) | 224 (9.3) | **<0.001** |
| LV Ejection Fraction |  |  | **<0.001** |
| *Normal (≥50%)* | 403 (51.0) | 1417 (59.1) |  |
| *Mildly reduced (40 to <50%)* | 72 (9.1) | 230 (9.6) |  |
| *Moderately reduced (30 to <40%)* | 136 (17.2) | 419 (17.5) |  |
| *Severely reduced (<30%)* | 179 (22.7) | 333 (13.9) |  |
| LV Diastolic Dysfunction | 677 (85.7) | 1903 (79.3) | **<0.001** |
| Dilated RV | 178 (22.5) | 332 (13.8) | **<0.001** |
| RV Dysfunction | 230 (29.1) | 429 (17.9) | **<0.001** |
| Pulmonary Hypertension | 332 (42.0) | 733 (30.6) | **<0.001** |
| Dilated Inferior Vena Cava | 99 (12.5) | 168 (7.0) | **<0.001** |
| Pericardial Effusion | 47 (5.9) | 118 (4.9) | 0.297 |

Continuous variables are expressed as mean ± standard deviation. The remaining categorical variables are expressed in terms of n (%). Comparisons between groups was performed using either a t-test or chi-square test. Variables with a p <0.10 (bold) were selected further inclusion in machine-learning-based variable selection.
